# Concordance with urgent referral guidelines in patients presenting with any of six ‘alarm’ features of possible cancer: a retrospective cohort study using linked primary care records

**DOI:** 10.1136/bmjqs-2021-013425

**Published:** 2021-10-04

**Authors:** Bianca Wiering, Georgios Lyratzopoulos, Willie Hamilton, John Campbell, Gary Abel

**Affiliations:** 1 University of Exeter Medical School, College of Medicine and Health, University of Exeter, Exeter, UK; 2 Epidemiology of Cancer Healthcare and Outcomes Group, Department of Behavioral Science and Health, Institute of Epidemiology and Health Care, University College London, London, UK

**Keywords:** primary care, health services research, health policy, healthcare quality improvement, general practice

## Abstract

**Background:**

Clinical guidelines advise GPs in England which patients warrant an urgent referral for suspected cancer. This study assessed how often GPs follow the guidelines, whether certain patients are less likely to be referred, and how many patients were diagnosed with cancer within 1 year of non-referral.

**Methods:**

We used linked primary care (Clinical Practice Research Datalink), secondary care (Hospital Episode Statistics) and cancer registration data. Patients presenting with haematuria, breast lump, dysphagia, iron-deficiency anaemia, post-menopausal or rectal bleeding for the first time during 2014–2015 were included (for ages where guidelines recommend urgent referral). Logistic regression was used to investigate whether receiving a referral was associated with feature type and patient characteristics. Cancer incidence (based on recorded diagnoses in cancer registry data within 1 year of presentation) was compared between those receiving and those not receiving referrals.

**Results:**

48 715 patients were included, of which 40% (n=19 670) received an urgent referral within 14 days of presentation, varying by feature from 17% (dysphagia) to 68% (breast lump). Young patients (18–24 vs 55–64 years; adjusted OR 0.20, 95% CI 0.10 to 0.42, p<0.001) and those with comorbidities (4 vs 0 comorbidities; adjusted OR 0.87, 95% CI 0.80 to 0.94, p<0.001) were less likely to receive a referral. Associations between patient characteristics and referrals differed across features: among patients presenting with anaemia, breast lump or haematuria, those with multi-morbidity, and additionally for breast lump, more deprived patients were less likely to receive a referral. Of 29 045 patients not receiving a referral, 3.6% (1047) were diagnosed with cancer within 1 year, ranging from 2.8% for rectal bleeding to 9.5% for anaemia.

**Conclusions:**

Guideline recommendations for action are not followed for the majority of patients presenting with common possible cancer features. A significant number of these patients developed cancer within 1 year of their consultation, indicating scope for improvement in the diagnostic process.

## Introduction

In 2015, the US Institute of Medicine report ‘Improving Diagnosis in Health Care’ highlighted the need to improve the quality and safety of diagnosis.^1^ It noted that ‘there are few clinical practice guidelines available for diagnosis’, contrasting this situation with the large number of guidelines aimed at improving the management of patients with a diagnosed illness. Guidelines for the management of patients with symptoms of possible cancer, which have been introduced in some health systems such as the English National Health Service (NHS), represent an important exception warranting investigation.^2–4^

The period between the patient’s first symptomatic presentation and cancer diagnosis is important, as shorter diagnostic intervals may contribute to an earlier stage at diagnosis and better patient experience.^5–8^ In order to improve the quality of diagnostic care and reduce delay, guidelines introduced in England in 2000 recommend that patients who present with certain features of possible cancer should be referred to hospital services by their GP for specialist assessment within 2 weeks. Relevant ‘red-flag’ features for which urgent referrals are recommended were defined by the National Institute for Health and Care Excellence (NICE) initially in 2005^3 4^ and updated in 2015 mainly in order to lower the cancer risk threshold for inclusion of features to 3%.^9^ The guidelines include presenting features, which cover clinical signs such as breast lump, test results such as iron-deficiency anaemia, and symptoms such as haematuria.

Guidelines can only ever be as effective as the degree to which they are implemented. The implementation of the Two Week Wait guidelines has been associated with a continuous increase in the number of patients (with or without cancer) who are investigated expeditiously, and in improvements in care outcomes in patients with cancer.^10–14^ While this evidence indicates that the Two Week Wait guidelines are helping to improve diagnostic processes and outcomes in England, the extent to which they are adhered to is unknown. Other evidence suggests that there is some variation in the quality of diagnostic care in cancer patients initially presenting to primary care,^15–18^ the use of endoscopic investigations between GP practices, and the proportion of cancer patients who were diagnosed after a Two Week Wait referral.^16 19–21^ Furthermore, previous observed inequalities in stage at diagnosis, referral interval and survival^22–27^ may suggest an association between certain patient characteristics (such as age, gender, socioeconomic status and ethnicity) and the risk of guideline non-adherence.

In principle, guideline-discordant referral behaviour may reflect accurate clinical judgement resulting in no adverse clinical outcomes for patients. How often this may be the case is, however, unknown, as studies thus far typically examined selected patients, such as from case series of malpractice claims including patients known to have been harmed.^28–30^ There is little evidence about the outcomes of non-adherence in population-based data. In this paper we focus on concordance with referral guidelines for patients presenting with features of possible cancer. Our research questions are:

1. To what extent are feature-based recommendations about referrals for suspected cancer included in NICE guidelines not followed?
2. Are patient factors associated with guideline discordant/concordant care regarding referral decisions for suspected cancer?
3. What is the proportion of patients who were diagnosed with cancer within 1 year after guideline discordant non-referral?

## Methods

### Data source

The Clinical Practice Research Datalink (CPRD) is a large database of routinely collected UK primary care health records. In this study, we used CPRD GOLD data which covered approximately 7% of the UK population in 2014^31^ and is collected from general practices using the Vision clinical software system. It contains clinical and administrative primary care data including diagnoses, investigations and treatments. A subset of CPRD GOLD practices subscribe to allow linkage of data to other sources. The CPRD data were linked at patient level to outpatient Hospital Episode Statistics (HES) data providing information on all referrals to NHS hospitals or NHS funded treatment centres, plus National Cancer Registration and Analysis Service (NCRAS) data providing information on cancer diagnoses, and patients’ Index of Multiple Deprivation (IMD—an area based measure of socioeconomic status based on the postcode of a patient’s residence).^32^ CPRD linked data have been used extensively for cancer diagnostic studies.^33–39^ HES^40^ and NCRAS^41–43^ quality have been validated and CPRD maintain exacting standards regarding data quality and validation.^44 45^

### Study population

We studied patients presenting to primary care during 2014 and 2015 who presented with one of six features, as recorded in primary care notes: dysphagia, post-menopausal bleeding, iron-deficiency anaemia, rectal bleeding, haematuria, and female breast lump. For these presenting features NICE guidelines recommend urgent (i.e. Two Week Wait) referral for suspected cancer. Inclusion and exclusion criteria are shown in table 1. Age restrictions reflect those patients covered by the guidelines.

**Table 1:** Patient inclusion and exclusion criteria

Patient inclusion criteria		Patient exclusion criteria
Registered at a CPRD GOLD practice that subscribes to data linkage		Patients who consulted with the same feature of interest as the index consultation in the year before the index consultation‡
A consultation with a medical code or test result (in the case of iron-deficiency anaemia) corresponding to a feature of interest during 2014 or 2015*		
Minimum age of patient at presentation	Dysphagia – 18	
	Post-menopausal bleeding – 45†	
	Iron-deficiency anaemia – 60	
	Rectal bleeding – 50	
	Haematuria – 45	
	Breast lump – 30	
Continuous registration at the same general practice from a year before to a year after their first index consultation.‡		

This table was created by the authors.

*There were minor changes in the recommendations for patients presenting with iron-deficiency anaemia and haematuria between the original (2005) and updated (2015) guidance. Therefore, we have restricted analysis to patients where an urgent referral would have been recommended in both sets of guidelines. Specifically for anaemia, inclusion as a first presentation was based on the more stringent test values included in the 2005 NICE guidelines of a haemoglobin level ≤11 g/dL for men and ≤10 g/dL for women, plus a ferritin level <20 ng/mL and/or a mean red cell volume <80 fL, and age restriction of 60 years and over related to the 2015 NICE guidelines. For haematuria, the 2015 NICE guidelines advise referral only if there is no evidence of a urinary tract infection, or if haematuria recurs or persists after successful treatment of a urinary tract infection. As such, the first and/or second consultation where antibiotics were prescribed in the absence of referral were excluded. Third visits within 6 months of the first visit were included regardless of GP treatment and referral decision-making.

†Although the guidelines do not specify an age range for post-menopausal bleeding, we exclude patients under the age of 45 years due to the small number in our sample (n=30).

‡With the exception of patients who presented with haematuria, the index consultation was defined for each patient as the first consultation with one or more of the six features based on medical codes for five features, and test results for iron-deficiency anaemia. For some patients presenting with haematuria, the second or third visit was included as their index consultation instead of the first visit, but exclusion criteria were applied to their first visit.

CPRD, Clinical Practice Research Datalink; GP, general practitioner; NICE, National Institute for Health and Care Excellence.

### Patient and public involvement

A patient and public involvement group comprising five members met two times throughout the study period. The group was asked to reflect on the study design and research questions. Results, their meaning, and potential explanations were discussed at several stages. They contributed to identifying explanations for GP referral decision making and differences in referral level between patient groups.

### Data analysis

The index consultation was defined for each patient as the first consultation with one of the six features. For these patients, referrals were identified in the HES dataset. Referral in HES is linked to the date the referral was received by the hospital, and recorded as ‘Routine’, ‘Urgent’, ‘Two Week Wait’ or ‘Unknown’. As the NICE guidelines define an urgent referral as a referral to see a specialist within 2 weeks, an outcome of urgent referral was defined as a referral having been made in the 14 days after the index consultation with a recorded urgency of ‘Urgent’ or ‘Two Week Wait’. The choice for this definition was pragmatic, acknowledging that there may be administrative delays between a consultation occurring and a referral being made, and was supported by our data showing that most patients referred for an urgent assessment were referred within 2 weeks of the index consultation (online supplemental appendix A, figure A1). Existing feature code lists were used to identify presentations with features of interest. These code lists were developed using robust methods^46^ and have been used successfully in previous studies.^38 47^ Patient age, gender, comorbidities, and previous cancer diagnosis were extracted from CPRD. Age was treated as a categorical variable with an 18- to 24-year-old group, 10 year age groups between 25 and 84, and an 85 and older group. Comorbidities were conditions included in the Quality of Outcome Framework 2015/2016, using existing code lists applied to all CPRD data before a patient’s index consultation. A simple count of comorbidities was used, grouping those with four or more comorbidities together. National quintiles were used to define IMD groups.

10.1136/bmjqs-2021-013425.supp1 Supplementary data

After describing the proportion of patients receiving an urgent referral, we investigated which patient groups were more or less likely to receive an urgent referral using multilevel logistic regression. An initial main effects-only model included patient age, gender, deprivation level, number of comorbidities, feature type, previous history of cancer, and a random intercept for referring clinician and general practice to account for clustering of patients within referring clinicians within practices. Between clinician and practice variation is quantified using the odds ratio covering the 95% mid-range of practices calculated from the estimated random effect variances.^48^ Reference categories were based on the highest number of patients with all genders and features represented. Interaction terms between patient characteristics and feature type were investigated individually with all significant interactions retained in the final model. We excluded 18 cases due to missing data on deprivation level. Significance of categorical variables was tested with a joint Wald test. The study power calculation assumed at least 8000 patients presenting with each feature, which would provide 90% power (p=0.05) to see a 4% absolute change from 25% discordant care in groups comprising 20% of the sample for any one feature (for example, 29% discordant care in the most deprived fifth of patients compared with 25% in others).

Finally, we investigated how many patients were diagnosed with cancer within 1 year of their index consultation by whether they received an urgent referral or not. Cancer incidence was based on the presence of a tumour in the NCRAS data with an International Classification of Diseases, 10th revision (ICD-10) invasive neoplasm code (C00 to C97 but excluding non-melanoma skin cancer, C44) and a date of diagnosis within 1 year of the index consultation.

### Sensitivity analyses

We performed five sensitivity analyses. First, we repeated the analysis excluding urgent referrals and solely focusing on Two Week Wait ones, to assess whether including urgent referrals had an impact on the findings. Second, we examined the impact of our restriction that referrals must occur within 2 weeks of the index consultation by expanding this window to 90 days. Third, the main analysis was repeated after also adjusting for ethnicity (extracted from HES; coded as white, black, Asian, mixed, and other ethnicity), which was not included in our main analysis due to a considerable amount of missing data (38%). Fourth, in addition to referrals captured in HES we also considered referrals recorded in CPRD. Where a record of a referral flagged as ‘Two Week Wait’, ‘Red flag’ or ‘Urgent’ in either CPRD or HES was made within 2 weeks of first presentation, the patient was considered to have had an urgent referral. This sensitivity analysis allows for the possibility that GPs made referrals which were not recorded in HES (either because changes were made to the referral after it was made or because the referral was not captured by HES, for example, for an investigation outside of the outpatient setting). Fifth, a sensitivity analysis was performed including neoplasm in situ (ICD-10 codes D00 to D48) in addition to invasive neoplasms. All analyses were conducted using the statistical software Stata v14.2.^49^

## Results

### Patient characteristics

There were 48 715 index consultations by patients with a feature of interest where a Two Week Wait referral would have been recommended (table 2). Among these patients, the most common presenting features were breast lump (33%) and rectal bleeding (27%). The mean age of the included patients was 60.4 years (range 18–104; SD 15.6). However, age ranges varied by feature, with the lowest mean age of 49.5 years (range 30–104 years; SD 13.7) for patients with breast lump and the highest mean age of 77.9 years for patients with anaemia (range 60–102; SD 9.1). Most patients had at least one comorbidity (80%). Patients who lived in an area classed as the lowest quintile (least deprived) of the IMD were over-represented (26% vs an expected 20%).

**Table 2:** Patient characteristics per feature

	Anaemia (n=1268)	Rectal bleeding (n=13 067)	Dysphagia (n=8197)	Breast lump (n=16 118)	Haematuria (n=6529)	Post-menopausal bleeding (n=3536)	Total (n=48 715)
	N (%)	N (%)	N (%)	N (%)	N (%)	N (%)	
Age							
18 to 24 years	–	–	191 (2.3%)	–	–	–	191 (0.4%)
25 to 34 years	–	–	404 (4.9%)	1825 (11.3%)	–	–	2229 (4.6%)
35 to 44 years	–	–	733 (8.9%)	4798 (29.8%)	–	–	5531 (11.4%)
45 to 54 years	–	2355 (18.0%)	1308 (16.0%)	5072 (31.5%)	1107 (17.0%)	1010 (28.6%)	10 852 (22.3%)
55 to 64 years	103 (8.1%)	3815 (29.2%)	1521 (18.6%)	1883 (11.7%)	1305 (20.0%)	1463 (41.4%)	10 090 (20.7%)
65 to 74 years	350 (27.6%)	3509 (26.9%)	1660 (20.3%)	1432 (8.9%)	1826 (28.0%)	658 (18.6%)	9435 (19.4%)
75 to 84 years	481 (37.9%)	2468 (18.9%)	1513 (18.5%)	791 (4.9%)	1629 (25.0%)	313 (8.9%)	7195 (14.8%)
85 or older	334 (26.3%)	920 (7.0%)	867 (10.6%)	317 (2.0%)	662 (10.1%)	92 (2.6%)	3192(6.6%)
Gender (female)	709 (55.9%)	6547 (50.1%)	4519 (55.1%)	16 118 (100%)	2223 (34.1%)	3536 (100%)	33 652 (69.1%)
IMD							
1 (least deprived)	249 (19.6)	3475 (26.6%)	1864 (22.7%)	4368 (27.1%)	1715 (26.3%)	1001 (28.3%)	12 672 (26.0%)
2	276 (21.8%)	2996 (22.9%)	1727 (21.1%)	3491 (21.7%)	1548 (23.7%)	795 (22.5%)	10 833 (22.2%)
3	277 (21.9%)	2733 (20.9%)	1786 (21.8%)	3284 (20.4%)	1390 (21.3%)	718 (20.3%)	10 188 (20.9%)
4	272 (21.5%)	2193 (16.8%)	1524 (18.6%)	2737 (17.0%)	1086 (16.6%)	529 (15.0%)	8341 (17.1%)
5 (most deprived)	192 (15.1%)	1664 (12.7%)	1294 (15.8%)	2232 (13.9%)	789 (12.1%)	492 (13.9%)	6663 (13.7%)
Ethnicity							
White	796 (62.8%)	6392 (48.9%)	3980 (48.6%)	10 535 (65.4%)	4130 (63.3%)	2249 (63.6%)	28 082 (57.7%)
Black	13 (1.0%)	116 (0.9%)	54 (0.7%)	254 (1.6%)	58 (0.9%)	61 (1.7%)	556 (1.1%)
Asian	31 (2.4%)	222 (1.7%)	182 (2.2%)	364 (2.3%)	93 (1.4%)	95 (2.7%)	987 (2.0%)
Mixed	3 (0.2%)	28 (0.2%)	24 (0.3%)	112 (0.7%)	14 (0.2%)	12 (0.3%)	193 (0.4%)
Other	12 (1.0%)	78 (0.6%)	58 (0.7%)	184 (1.1%)	38 (0.6%)	43 (1.2%)	413 (0.9%)
Comorbidities							
0	76 (6.0%)	2164 (16.6%)	1355 (16.5%)	4668 (29.0%)	927 (14.2%)	588 (16.6%)	9778 (20.1%)
1	175 (13.8%)	2991 (22.9%)	1690 (20.6%)	4174 (25.9%)	1332 (20.4%)	923 (26.1%)	11 285 (23.2%)
2	258 (20.4%)	2965 (22.7%)	1765 (21.5%)	3598 (22.3%)	1479 (22.7%)	808 (22.9%)	10 873 (22.3%)
3	265 (20.9%)	2220 (17.0%)	1364 (16.6%)	2170 (13.5%)	1249 (19.1%)	629 (17.8%)	7897 (16.2%)
≥4	494 (39.0%)	2727 (20.9%)	2023 (24.7%)	1508 (9.4%)	1542 (23.6%)	588 (16.6%)	8882 (18.2%)

This table was created by the authors.

IMD, Index of Multiple Deprivation.

### Urgent referrals

Overall, 40% (n=19 670) of patients received an urgent referral within 2 weeks of visiting the GP. The percentage of urgent referrals varied greatly by presenting feature, ranging from 17% (n=1384) for patients with dysphagia to 68% (n=11 007) for patients with breast lump (table 3).

**Table 3:** Type of referrals given to patients within 2 weeks after visiting the GP with one or more of the six features

Features	Number of patients who did not receive an urgent referral	Number of patients who did receive an urgent referral	Referral types*	
			Two Week Wait referrals	Urgent referrals
	N (%)	N (%)	N (%)*	N (%)*
Anaemia (n=1268)	1007 (79.4%)	261 (20.6%)	176 (13.9%)	85 (6.7%)
Rectal bleeding (n=13 067)	10 752 (82.3%)	2315 (17.7%)	1427 (10.9%)	888 (6.8%)
Dysphagia (n=8197)	6813 (83.1%)	1384 (16.9%)	990 (12.1%)	394 (4.8%)
Breast lump (n=16 118)	5111 (31.7%)	11 007 (68.3%)	8052 (50.0%)	2955 (18.3%)
Haematuria†(n=6529)	4043 (61.9%)	2486 (38.1%)	1479 (22.7%)	1007 (15.4%)
Post-menopausal bleeding (n=3536)	1319 (37.3%)	2217 (62.7%)	1598 (45.2%)	619 (17.5%)
Total (n=48 715)	29 045 (59.6%)	19 670 (40.4%)	13 722 (28.2%)	5948 (12.2%)

This table was created by the authors.

*Patients may have received several referral types within 2 weeks. The table describes whether patients received a Two Week Wait referral, and if not, whether they received an urgent referral.

†For haematuria, the number of referrals is based on referrals within 2 weeks after the first visit, referrals within 2 weeks after the second visit if patients received urinary tract infection treatment the first time, and referrals within 2 weeks after the third visit if patients received treatment during their first and second visit.

GP, general practitioner.

### Associations between patient characteristics and urgent referrals

The main effects-only models showed evidence that age (p<0.001), feature type (p<0.001), and comorbidities (p<0.001) were associated with the probability of receiving an urgent referral (online supplemental appendix A, figure A2; table 4). Patients with breast lump were most likely to receive a referral (adjusted OR compared with rectal bleeding: 16.85, 95% CI 15.56 to 18.26). Younger age (adjusted OR 18–24 years vs 55–64 years: 0.20, 95% CI 0.10 to 0.42) and greater number of comorbidities (adjusted OR 4 comorbidities vs 0 comorbidities: 0.87, 95% CI 0.80 to 0.94) were associated with a lower chance of receiving an urgent referral (online supplemental appendix A, figure A2; table 4). There was substantial variation between both clinicians and practices, with the modelled OR covering the middle 95% of clinicians being 2.62, and practice being 1.62. The final model, including interactions between feature and patient factors, indicated that the association between patient characteristics and urgent referrals was highly dependent on the patient’s presenting features. Although there was no evidence for an average effect of deprivation across all features, there was evidence that patients living in more deprived neighbourhoods were more likely to be referred urgently if they presented with haematuria, post-menopausal bleeding, or rectal bleeding, though the opposite was true for women presenting with breast lump (online supplemental appendix A, figure A3(a)). Furthermore, patients presenting with anaemia, breast lump or haematuria were less likely to receive an urgent referral if they had a higher number of comorbidities, though the gradient was stronger in patients with anaemia than for other features (online supplemental appendix A, figure A3(b)). Finally, although the association between age and urgent referral was similar for all patients, the size of age variation was greater for patients presenting with dysphagia and breast lump (online supplemental appendix A, figure A3(c)). However, this largely reflected the larger age range covered by referral guidelines for those features. Sensitivity analyses excluding the 58% of patients who received an urgent as opposed to Two Week Wait referral (online supplemental appendix A, table A1), or including ethnicity (online supplemental appendix A, table A2), showed similar results. The sensitivity analysis for urgent referrals occurring within 90 days of presentation showed that 44% (as opposed to 40% in the main analysis) of patients received a referral, and showed similar variation by age, feature type and number of comorbidities to the main analysis (online supplemental appendix A, table A3). Finally, the sensitivity analysis including urgent referrals recorded in either HES or CPRD within 2 weeks of the index consultation identified a further 3503 patients receiving a referral, indicating that 48% of patients received an urgent referral (online supplemental appendix A, table A4).

**Table 4:** Associations between patient characteristics and urgent referrals received within 2 weeks after visiting the GP (main analysis)

	Unadjusted OR	Lower 95% CI	Upper 95% CI	P value	OR	Lower 95% CI	Upper 95% CI	P value
Features								
Anaemia	1.17	1.01	1.37	<0.001	1.14	0.97	1.33	<0.001
Rectal bleeding	Ref	Ref	Ref		Ref	Ref	Ref	
Dysphagia	0.91	0.84	0.99		1.01	0.93	1.09	
Breast lump	12.15	11.41	12.94		16.85	15.56	18.26	
Haematuria	3.29	3.05	3.54		3.29	3.05	3.54	
Post-menopausal bleeding	9.57	8.74	10.48		9.93	9.01	10.94	
Age								
18 to 24 years	0.08	0.04	0.16	<0.001	0.20	0.10	0.42	<0.001
25 to 34 years	1.41	1.28	1.56		0.37	0.33	0.41	
35 to 44 years	2.25	2.10	2.42		0.58	0.53	0.64	
45 to 54 years	1.47	1.39	1.56		0.80	0.75	0.86	
55 to 64 years	Ref	Ref	Ref		Ref	Ref	Ref	
65 to 74 years	0.90	0.84	0.96		1.11	1.04	1.20	
75 to 84 years	0.83	0.78	0.89		1.20	1.11	1.30	
85 or older	0.64	0.58	0.71		0.96	0.86	1.07	
Sex (female)	3.06	2.92	3.21	<0.001	1.03	0.97	1.10	0.365
IMD								
1 (least deprived)	Ref	Ref	Ref	0.775	Ref	Ref	Ref	0.246
2	0.98	0.93	1.05		1.01	0.94	1.09	
3	1.03	0.96	1.10		1.05	0.98	1.13	
4	1.00	0.93	1.08		1.09	1.01	1.18	
5 (most deprived)	1.00	0.92	1.08		1.06	0.97	1.17	
Comorbidities								
0	Ref	Ref	Ref	<0.001	Ref	Ref	Ref	<0.001
1	0.90	0.85	0.95		0.99	0.92	1.06	
2	0.85	0.80	0.90		0.97	0.91	1.04	
3	0.74	0.69	0.79		0.89	0.82	0.96	
≥4	0.60	0.56	0.64		0.87	0.80	0.94	
Previous history of cancer	–	–	–		0.90	0.82	0.99	0.035

This table was created by the authors.

GP, general practitioner; IMD, Index of Multiple Deprivation.

### Cancer diagnoses

Of the 19 670 patients who received an urgent referral, 1950 (9.9%) went on to be diagnosed with cancer within 1 year. Of the 29 045 patients who did not receive an urgent referral, 1047 (3.6%) patients were diagnosed with cancer within 1 year. As a result, 35% of all patients diagnosed with cancer in the year after consulting their GP with one of the six features had not received a recorded urgent referral within 2 weeks of their index consultation (online supplemental appendix A, table A5). The percentage of patients diagnosed with cancer without having received a referral varied between features, from 2.8% for rectal bleeding to 9.5% for patients with iron-deficiency anaemia (table 5; Online supplemental appendix A, figure A4). The sensitivity analysis, which included urgent referrals captured by either CPRD or HES, found that 3.2% of patients who did not receive a referral were diagnosed with cancer within 1 year of their index consultation (online supplemental appendix A, table A6). The sensitivity analysis, including both invasive neoplasms and neoplasms in situ, showed similar results with 4.2% of patients diagnosed with either type of neoplasm within 1 year (compared with 3.6% for invasive neoplasms and HES recorded referrals alone) (online supplemental appendix A, table A7). The percentage of patients diagnosed with cancer of a specific site within 1 year of not receiving an urgent referral was low for most cancers (table 5), with the exceptions of colorectal cancer for patients with iron-deficiency anaemia (5.5%), breast cancer for patients with breast lump (3.5%), and uterine cancer for patients with post-menopausal bleeding (2.9%).

**Table 5:** Main cancer diagnoses within 1 year of the index GP visit

Features		Main cancer types for patients who did not receive an urgent referral			Main cancer types for patients who did receive an urgent referral		
		Cancer type	N	Percentage of patients diagnosed with cancer*	Cancer type	N	Percentage of patients diagnosed with cancer*
Anaemia	1	Colorectal cancer	55	5.5%	Colorectal cancer	44	16.9%
	2	Oesophagogastric cancer	12	1.2%	Oesophagogastric cancer	5	1.9%
	3	Breast cancer	7	0.7%	Prostate cancer	3	1.1%
		Total number of diagnoses†	98	9.7%	Total number of diagnoses†	64	24.5%
		Total number of patients diagnosed with cancer	96	9.5%	Total number of patients diagnosed with cancer	62	23.8%
Rectal bleeding	1	Colorectal cancer	159	1.5%	Colorectal cancer	148	6.4%
	2	Prostate cancer	41	0.4%	Breast cancer	11	0.5%
	3	Breast cancer	21	0.2%	Anal cancer	10	0.4%
		Total number of diagnoses†	316	2.9%	Total number of diagnoses†	205	8.9%
		Total number of patients diagnosed with cancer	299	2.8%	Total number of patients diagnosed with cancer	200	8.6%
Dysphagia	1	Oesophagogastric cancer	108	1.6%	Oesophagogastric cancer	53	3.8%
	2	Breast cancer	19	0.3%	Lip, oral cavity and pharynx cancer	10	0.7%
	3	Lung cancer	14	0.2%	Lung cancer	7	0.5%
		Total number of diagnoses†	212	3.1%	Total number of diagnoses†	89	6.4%
		Total number of patients diagnosed with cancer	209	3.1%	Total number of patients diagnosed with cancer	87	6.3%

Breast lump	1	Breast cancer	179	3.5%	Breast cancer	1138	10.3%
	2	Melanoma	4	0.1%	Colorectal cancer	9	0.1%
	3	Colorectal cancer	2	0.0%	Lung cancer	6	0.1%
		Total number of diagnoses†	194	3.8%	Total number of diagnoses†	1184	10.8%
		Total number of patients diagnosed with cancer	191	3.7%	Total number of patients diagnosed with cancer	1162	10.6%
Haematuria	1	Bladder cancer	66	1.6%	Bladder cancer	139	5.6%
	2	Prostate cancer	54	1.3%	Prostate cancer	54	2.2%
	3	Kidney cancer	24	0.6%	Kidney cancer	40	1.6%
		Total number of diagnoses†	209	5.2%	Total number of diagnoses†	289	11.6%
		Total number of patients diagnosed with cancer	200	5.0%	Total number of patients diagnosed with cancer	268	10.8%
Post-menopausal bleeding	1	Uterine cancer	38	2.9%	Uterine cancer	134	6.0%
	2	Breast cancer	5	0.4%	Ovarian cancer	11	0.5%
	3	Ovarian cancer	2	0.2%	Breast cancer	9	0.4%
		Total number of diagnoses†	53	4.0%	Total number of diagnoses†	179	8.1%
		Total number of patients diagnosed with cancer	52	3.9%	Total number of patients diagnosed with cancer	171	7.7%

This table was created by the authors.

*Percentage of cancer diagnoses compared with the number of patients who received or did not receive an urgent referral.

†Some patients received more than one separate cancer diagnosis within 1 year of their GP visit with a feature of interest.

GP, general practitioner.

## Discussion

### Statement of principal findings

Six out of 10 patients presenting to primary care with a high-risk feature of possible cancer did not receive an urgent referral in the 14 days after presentation, despite this being a guideline-recommended action. Urgent referral frequency varied by feature, with patients with breast lump receiving the highest percentage of referrals and patients with dysphagia the lowest. Younger patients, and those with comorbidities were less likely to receive an urgent referral. Associations between patient characteristics and urgent referrals differed by feature. More deprived women with breast lump, and patients with anaemia, breast lump, or haematuria and multi-morbidity were less likely to receive a referral; 3.6% of patients who did not receive an urgent referral were diagnosed with cancer within 1 year, this percentage varying between 2.8% for rectal bleeding and 9.5% for iron-deficiency anaemia.

### Strengths and limitations

We used a large, longitudinal, validated linked dataset which has been used extensively for cancer diagnostic studies,^10 50 51^ enabling important insights into patients’ journeys through the healthcare system. However, there are limitations. First, as an urgently referred patient would usually be investigated in an outpatient setting, the HES dataset comprised outpatient hospital data. Consequently, patients referred and admitted to hospital or directed to an emergency department will not have been identified even though timely action was taken. However, the number of such patients is likely small for the studied presenting features, especially given the substantial decrease in the number of cancers diagnosed following emergency GP referrals in the last decade.^13^ Second, identification of index consultations with one of the six features was based on medical codes in patient records. Some patients may have been missed because the feature was only recorded in inaccessible ‘free text’ (which is not available to researchers to avoid de-anonymisation) or because the feature was not recorded at all. However, CPRD studies of free-text data suggest it usually only confirms coded entries.^52^ Consequently, some patients with features of interest may not have been included in the study. However, those who were included almost certainly had the feature, and thus a Two Week Wait referral would have been recommended. Third, inclusion of patients was limited to 2014 and 2015 as cancer registry data were only available up to and including 2016 at the time of data extraction. However, it is unlikely that this will have affected results, as the guidelines had been in use for a number of years, and analyses were restricted to patients where an urgent referral would have been recommended in both the 2005 and 2015 guidance. Fourth, we excluded patients with haematuria who were treated for possible urinary tract infection (unless it was not the last visit or a third consultation within 6 months) on the basis of prescriptions for two of the most common first-line antibiotics for urinary tract infection. A small number of patients will have been prescribed different antibiotics and been included in error. Fifth, although the study showed that many patients were diagnosed with cancer after not receiving an urgent referral after presenting with a suspected cancer feature, we were unable to give insight into the potential impact of non-referral on cancer stage. Finally, we note that although for three features we exceeded our planned sample size, the number of patients presenting with iron-deficiency anaemia or post-menopausal bleeding was significantly less than planned.

### Comparison with existing evidence and meaning of the study

Previous studies have reported that the number of GP consultations before referral varied by cancer type.^53^ It appears that GPs are less likely to suspect cancer for some features compared with others. This may be partially explained by the greater likelihood of some features being caused by other explanations than cancer. However, the risk of cancer with all these features is always low in absolute terms, with iron-deficiency anaemia having the highest positive predictive value of the six for cancer (for men over 60 years with a haemoglobin <11 g/dL and features of iron deficiency, the positive predictive value is 13%^54^). The decision to refer may be influenced by factors other than guidelines, such as GPs’ symptom interpretation,^55^ additional presenting features supporting a different diagnosis, and clinical intuition,^56^ but also by how local health services are organised.^20^ Variation in referral has been shown to be partly attributable to Clinical Commissioning Groups (CCGs) and Acute Hospital Trusts.^20^ This is supported by qualitative research suggesting that GPs are hesitant to refer or even feel pressured by CCGs to not refer due to resource pressures.^57^ Additionally, although probably only responsible for a small proportion of non-referrals, there are a number of other factors which may affect whether a referral was made or recorded (box 1). Regardless of the GPs’ reasons for referral or non-referral, our study shows that GPs often made the right decision regarding referral for their patients. Given the proportion of patients going on to be diagnosed with cancer was considerably higher in those receiving an urgent referral than those who did not, we can conclude that GP referral decision-making is not without value. However, given the number of patients diagnosed with cancer after non-referral, we may question whether clinical judgement is good enough: 5.5% of patients with anaemia not receiving an urgent referral were diagnosed with colorectal cancer within 1 year, 3.5% of women presenting with breast lump who did not receive a referral were diagnosed with breast cancer, and 2.9% who presented with post-menopausal bleeding were diagnosed with uterine cancer. In these patients it can be argued that guideline-discordant decision-making may have resulted in a missed opportunity to diagnose early. Better adherence to the guidelines may therefore be important in order to increase detection rate, even for alarm features with already high urgent referral rates.

Box 1 **Mechanisms affecting referral**

Although we expect that it only affects a small number of patients, there are a number of mechanisms besides GP referral decision-making which may potentially influence whether an urgent referral was made or recorded:

- Patients were admitted to hospital via emergency admission
- The referral was not accepted by the hospital. (This should be captured by the sensitivity analysis including Clinical Practice Research Datalink (CPRD) referrals)
- The patient refused to be referred
- A downgrade of the urgency level of the referral was requested by the hospital. (This should still be captured in the CPRD sensitivity analysis)
- Index consultation took place with out-of-hours practice services
- Variations in local guidelines for referral. (For most patients this should be captured in either the sensitivity analysis including CPRD referrals or the sensitivity analysis including referrals made up to 90 days after presentation)
- The patient received a related referral before first presentation, which affected the decision to refer

Our finding that younger patients were less likely to receive an urgent referral reflects earlier research reporting that younger patients typically experience longer diagnostic timelines.^53^ The present study also offers new insights into how multi-morbidity may affect diagnostic timeliness. Although there are suggestions that more contact with health services can shorten diagnostic intervals, a growing body of evidence suggests that multi-morbidity can also prolong diagnostic intervals.^15 58–61^ Given our findings, it may be these prolonged intervals arise, in part, due to a lower likelihood of receiving an urgent referral. Additionally, research suggests that multi-morbidity is associated with decreased use of specialist investigations.^62^ This may explain the strong multi-morbidity gradient observed for patients with anaemia where urgent referral would lead to an invasive test. Future research investigating how multi-morbidity affects GP referral decision-making for potential cancer features may help target improvement efforts.

Although, on average, deprivation was not associated with urgent referrals, more deprived patients with haematuria, rectal or post-menopausal bleeding were more likely to receive a referral. Although we can only speculate, this finding could be explained by earlier research suggesting that deprived patients are more likely to delay presentation,^63 64^ resulting in more serious potential cancer features, increasing the chance of an urgent referral. On the other hand, more deprived women presenting with breast lump were less likely to receive an urgent referral. As few women with breast cancer delay presentation,^63^ the association between deprivation and referral is likely to reflect differences post-presentation. Patients with a higher socioeconomic status tend to be more effectively able to communicate their symptoms and concerns,^65 66^ while GPs’ communication tends to be more patient-centred with less deprived patients.^67^ This may potentially result in closer alignment in perceptions of symptom significance^68^ and influence GPs’ decision to refer.

We have identified patient groups who may be at risk of longer diagnostic timelines. GPs may be less likely to refer patients when their age,^69^ or alternative medical explanations, suggest a lower risk of cancer. However, guidelines incorporate patient age and thus recommended action is still appropriate for those age groups.

Clinical practice guidelines have been shown to improve treatment quality for a range of conditions and could also help to improve the quality of the diagnostic process. However, our study shows that recommendations for the assessment of patients with features of possible cancer are not always followed. Stricter adherence to the guidelines and increased awareness of patient groups especially at risk of long diagnostic timelines may help improve early diagnosis and ultimately cancer survival rates. Due to the potential impact of regional health services, interventions to reduce guideline discordant behaviour may have more impact if they do not just focus on GPs and individual practices, but also on local diagnostic service provision.

## Data Availability

Data may be obtained from a third party and are not publicly available. Routinely collected patient electronic health data was provided by CPRD. Access to data from CPRD is subject to a licence agreement and protocol approval from the Independent Scientific Advisory Committee (ISAC).
