# Supplementary material for: Concordance with urgent referral guidelines in patients presenting with any of six ‘alarm’ features of possible cancer: a retrospective cohort study using linked primary care records
Source: BMJ Qual Saf. 2021 Oct 4;31(8):579–89. doi: 10.1136/bmjqs-2021-013425 (PMC9304100; doi:10.1136/bmjqs-2021-013425)
Supplement: Supplementary data [file bmjqs-2021-013425supp001.pdf]

Appendix A.

Table A1. Associations between patient characteristics and two week wait referrals received within two weeks of visiting the GP (sensitivity analysis considering only referrals flagged as "two week wait"). This table was created by the authors.

	Unadjusted odds ratio	Lower 95% confidence interval	Upper 95% confidence interval	P	Odds ratio	Lower 95% confidence interval	Upper 95% confidence interval	P
Features								
Anaemia	1.35	1.12	1.62	<0.001	1.28	1.06	1.54	<0.001
Rectal bleeding	*Ref*	*Ref*	*Ref*		*Ref*	*Ref*	*Ref*	
Dysphagia	1.12	1.02	1.23		1.24	1.13	1.36	
Breast lump	12.91	11.98	13.90		18.05	16.43	19.83	
Haematuria	2.97	2.72	3.25		2.94	2.68	3.23	
Post-menopausal bleeding	9.69	8.74	10.75		10.30	9.22	11.51	
Age								
18 to 24 years	0.06	0.02	0.16	<0.001	0.14	0.05	0.38	<0.001
25 to 34 years	1.36	1.21	1.53		0.36	0.32	0.42	
35 to 44 years	2.33	2.15	2.53		0.62	0.56	0.69	
45 to 54 years	1.52	1.42	1.63		0.83	0.76	0.90	
55 to 64 years	*Ref*	*Ref*	*Ref*		*Ref*	*Ref*	*Ref*	
65 to 74 years	0.92	0.85	0.99		1.18	1.08	1.28	
75 to 84 years	0.83	0.77	0.90		1.24	1.13	1.37	
85 or older	0.68	0.61	0.76		1.05	0.92	1.20	
Sex (Female)	3.32	3.13	3.52	<0.001	1.00	0.93	1.08	1.000
IMD								
1 (least deprived	*Ref*	*Ref*	*Ref*	0.744	*Ref*	*Ref*	*Ref*	0.122
2	0.97	0.91	1.04		0.99	0.91	1.07	
3	0.95	0.88	1.03		0.93	0.86	1.02	
4	0.98	0.90	1.07		1.05	0.96	1.16	
5 (most deprived)	0.96	0.87	1.06		0.97	0.87	1.09	
Comorbidities								
0	*Ref*	*Ref*	*Ref*	<0.001	*Ref*	*Ref*	*Ref*	0.008
1	0.88	0.82	0.94		0.97	0.90	1.05	
2	0.81	0.75	0.86		0.92	0.85	1.00	
3	0.72	0.67	0.78		0.88	0.80	0.96	
4 or more	0.60	0.56	0.65		0.87	0.79	0.96	
Previous history of cancer	-	-	-		0.89	0.80	0.99	0.030

Table A2. Associations between patient characteristics (including ethnicity) and urgent referrals received within two weeks after visiting the GP (sensitivity analysis including adjustment for ethnicity). This table was created by the authors.

	Unadjusted odds ratio	Lower 95% confidence interval	Upper 95% confidence interval	P	Odds ratio	Lower 95% confidence interval	Upper 95% confidence interval	P
Features								
Anaemia	1.17	1.01	1.37	<0.001	0.91	0.77	1.08	<0.001
Rectal bleeding	*Ref*	*Ref*	*Ref*		*Ref*	*Ref*	*Ref*	
Dysphagia	0.91	0.84	0.99		0.97	0.88	1.06	
Breast lump	12.15	11.41	12.94		14.77	13.36	16.33	
Haematuria	3.29	3.05	3.54		2.75	2.52	3.01	
Post-menopausal bleeding	9.57	8.74	10.48		8.80	7.79	9.95	
Age								
18 to 24 years	0.08	0.04	0.16	<0.001	0.20	0.08	0.52	<0.001
25 to 34 years	1.41	1.28	1.56		0.37	0.31	0.43	
35 to 44 years	2.25	2.10	2.42		0.60	0.53	0.68	
45 to 54 years	1.47	1.39	1.56		0.82	0.75	0.90	
55 to 64 years	*Ref*	*Ref*	*Ref*		*Ref*	*Ref*	*Ref*	
65 to 74 years	0.90	0.84	0.96		1.03	0.94	1.12	
75 to 84 years	0.83	0.78	0.89		1.06	0.97	1.17	
85 or older	0.64	0.58	0.71		1.00	0.88	1.13	
Sex (Female)	3.06	2.92	3.21	<0.001	1.03	0.96	1.11	0.409
IMD								
1 (least deprived	*Ref*	*Ref*	*Ref*	0.775	*Ref*	*Ref*	*Ref*	0.792
2	0.98	0.93	1.05		0.95	0.87	1.04	
3	1.03	0.96	1.10		0.96	0.88	1.06	
4	1.00	0.93	1.08		0.99	0.90	1.10	
5 (most deprived)	1.00	0.92	1.08		0.95	0.85	1.07	
Comorbidities								
0	*Ref*	*Ref*	*Ref*	<0.001	*Ref*	*Ref*	*Ref*	<0.001
1	0.90	0.85	0.95		0.95	0.87	1.04	
2	0.85	0.80	0.90		0.88	0.80	0.96	
3	0.74	0.69	0.79		0.76	0.69	0.83	
4 or more	0.60	0.56	0.64		0.69	0.63	0.77	
Ethnicity								
White	*Ref*	*Ref*	*Ref*	0.180	*Ref*	*Ref*	*Ref*	0.864
Black	1.10	0.91	1.33		0.93	0.75	1.16	
Asian	0.89	0.77	1.03		0.95	0.80	1.11	
Mixed	1.20	0.87	1.64		0.88	0.62	1.26	

Other	1.14	0.92	1.43		1.02	0.80	1.31	
Previous history of cancer	-	-	-	-	0.85	0.76	0.95	0.004

Table A3. Associations between patient characteristics and urgent referrals received within 90 days of visiting the GP (sensitivity analysis considering referrals made up to 90 days after presentations rather than up to 14 days as in the main analysis). This table was created by the authors.

	Unadjusted odds ratio	Lower 95% confidence interval	Upper 95% confidence interval	P	Odds ratio	Lower 95% confidence interval	Upper 95% confidence interval	P
Features								
Anaemia	1.49	1.30	1.71	<0.001	1.43	1.25	1.64	<0.001
Rectal bleeding	*Ref*	*Ref*	*Ref*		*Ref*	*Ref*	*Ref*	
Dysphagia	0.90	0.84	0.97		0.99	0.92	1.07	
Breast lump	10.43	9.83	11.07		14.02	12.99	15.14	
Haematuria	2.32	2.16	2.48		2.31	2.15	2.48	
Post-menopausal bleeding	8.72	7.97	9.54		9.03	8.21	9.94	
Age								
18 to 24 years	0.07	0.04	0.14	<0.001	0.17	0.09	0.34	<0.001
25 to 34 years	1.51	1.37	1.67		0.42	0.37	0.47	
35 to 44 years	2.31	2.15	2.48		0.62	0.57	0.68	
45 to 54 years	1.42	1.34	1.50		0.80	0.74	0.85	
55 to 64 years	*Ref*	*Ref*	*Ref*		*Ref*	*Ref*	*Ref*	
65 to 74 years	0.91	0.85	0.96		1.11	1.04	1.19	
75 to 84 years	0.87	0.81	0.93		1.23	1.14	1.33	
85 or older	0.65	0.59	0.71		0.93	0.84	1.03	
Sex (Female)	3.01	2.87	3.15	<0.001	1.04	0.98	1.10	0.218
IMD								
1 (least deprived	*Ref*	*Ref*	*Ref*	0.578	*Ref*	*Ref*	*Ref*	0.138
2	0.98	0.92	1.04		1.00	0.93	1.07	
3	1.03	0.96	1.10		1.05	0.97	1.13	
4	1.02	0.95	1.09		1.09	1.01	1.18	
5 (most deprived)	1.02	0.94	1.10		1.07	0.98	1.18	
Comorbidities								
0	*Ref*	*Ref*	*Ref*	<0.001	*Ref*	*Ref*	*Ref*	0.026
1	0.90	0.85	0.95		0.99	0.92	1.06	
2	0.86	0.81	0.91		0.99	0.93	1.06	

3	0.76	0.71	0.81	0.91	0.85	0.98	
4 or more	0.64	0.60	0.68	0.91	0.84	0.99	
Previous history of cancer	-	-	-	0.91	0.83	1.00	0.047

Table A4. Urgent referral within two weeks of first presentation as captured by either HES or CPRD (sensitivity analysis considering urgent referrals recorded in either CPRD or HES). This table was created by the authors.

Symptom	No urgent referral in HES (%)	Urgent referral in HES (%)	No urgent referral in CPRD and HES (%)	Urgent referral in CPRD and HES (%)
	Main analysis using HES data		Sensitivity analysis using HES and CPRD data	
	N (%)	N (%)	N (%)	N (%)
Anaemia (n=1268)	1007 (79.4%)	261 (20.6%)	939 (74.1%)	329 (26.0%)
Rectal bleeding (n=13067)	10752 (82.3%)	2315 (17.7%)	10168 (77.8%)	2899 (22.2%)
Dysphagia (n=8197)	6813 (83.1%)	1384 (16.9%)	5431 (66.3%)	2766 (33.7%)
Breast lump (n=16118)	5111 (31.7%)	11007 (68.3%)	4471 (27.7%)	11647 (72.3%)
Haematuria (n=6604)	4043 (61.9%)	2486 (38.1%)	3507 (53.1%)	3097 (46.9%)
PMB (n=3536)	1319 (37.3%)	2217 (62.7%)	1101 (31.1%)	2435 (68.9%)
Total (n=48715 for HES; n= 48790 for CPRD and HES)	29045 (59.6%)	19670 (40.4%)	25617 (52.5%)	23173 (47.5%)

Table A5. Urgent referrals for patients diagnosed with cancer. This table was created by the authors.

Features	Patients with neoplasms excluding neoplasms in situ[1]	
	No urgent referral	Urgent referral
	N (%)	N (%)
Anaemia (n=158)	96 (60.8%)	62 (39.2%)
Rectal bleeding (n=499)	299 (59.9%)	200 (40.1%)
Dysphagia (n=296)	209 (70.6%)	87 (29.4%)
Breast lump (n=1353)	191 (14.1%)	1162 (85.9%)
Haematuria (n=468)	200 (42.7%)	268 (57.3%)
Post-menopausal bleeding (n=223)	52 (23.3%)	171 (76.7%)

[1]ICD-10-CM C codes excluding C44

Table A6. Cancer incidence in patients with and without urgent referral as captured by either HES or CPRD (sensitivity analysis considering urgent referrals recorded in either CPRD or HES). This table was created by the authors.

	Cancer incidence	
Symptom	No urgent referral in CPRD and HES (%*)	Urgent referral in CPRD and HES (%*)
Anaemia	79 (8.4%)	79 (24.0%)
Rectal bleeding	277 (2.7%)	222 (7.7%)
Dysphagia	140 (2.6%)	156 (5.6%)
Breast lump	134 (3.0%)	1,219 (10.5%)
Haematuria	153 (4.4%)	320 (10.3%)
PMB	39 (3.5%)	184 (7.6%)
Total	822 (3.2%)	2,180 (9.4%)

* Percentage of cancer diagnoses compared to the number of patients who received or did not receive an urgent referral

Table A7. Cancer and neoplasms in situ incidence in patients who did not receive an urgent referral (sensitivity analysis including neoplasms in situ). This table was created by the authors.

Features	No cancer diagnosis	Cancer diagnosis
	N (%)	N (%)
Anaemia (n=1007)	904 (89.8%)	103 (10.2%)
Rectal bleeding (n= 10752)	10417 (96.9%)	335 (3.1%)
Dysphagia (n= 6813)	6591 (96.7%)	222 (3.3%)
Breast lump (n=5111)	4906 (96.0%)	205 (4.0%)
Haematuria (n=4043)	3751 (92.8%)	292 (7.2%)
Post-menopausal bleeding (n=1319)	1264 (95.8%)	55 (4.2%)

Table A8. Urgent referrals for patients diagnosed with neoplasms, including neoplasms in situ. This table was created by the authors.

Features	Patients with neoplasms including neoplasms in situ[1]	
	No urgent referral	Urgent referral
	N (%)	N (%)
Anaemia (n=166)	103 (62.1%)	63 (38.0%)
Rectal bleeding (n=557)	335 (60.1%)	222 (39.9%)
Dysphagia (n=310)	222 (71.6%)	88 (28.4%)
Breast lump (n=1425)	205 (14.4%)	1220 (85.6%)
Haematuria (n=721)	292 (40.4%)	430 (59.6%)
Post-menopausal bleeding (n=229)	55 (24.0%)	174 (76.0%)

[1]ICD-10-CM C and D codes excluding C44

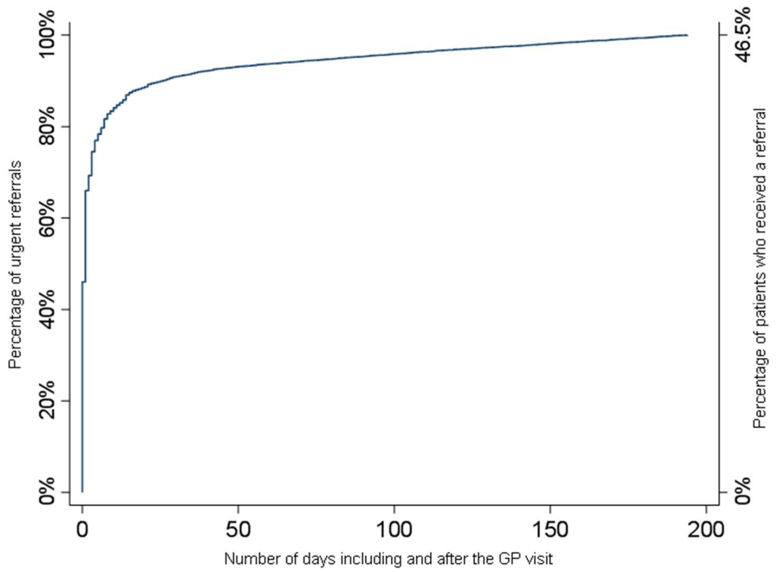

Figure A1. Cumulating urgent referrals within six months of the GP index visit. This figure was created by the authors.

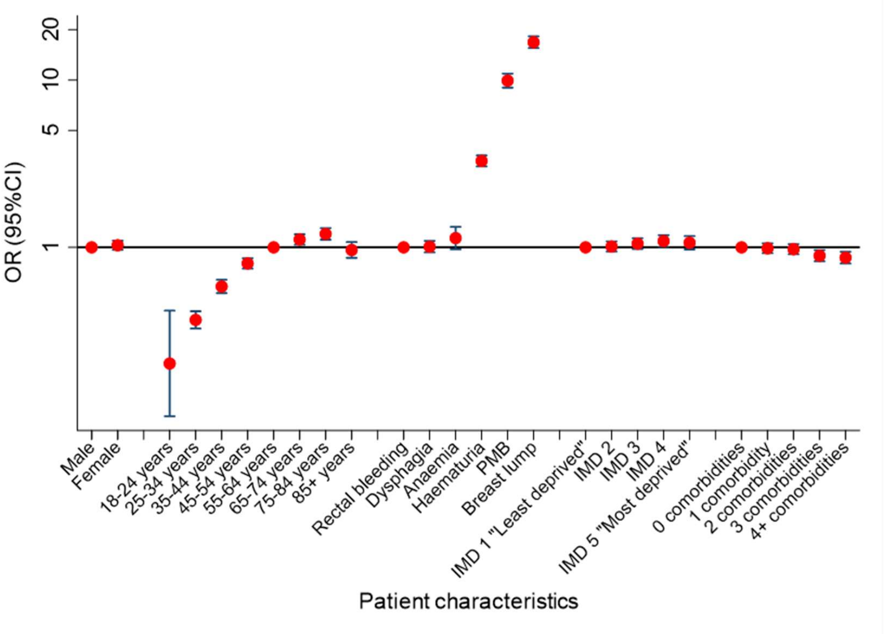

Figure A2. Associations between patient characteristics and receiving an urgent referral within two weeks of visiting the GP. This figure was created by the authors.

Figure A3: Associations between patient characteristics ((a) deprivation; (b) comorbidities; (c) age) and receiving an urgent suspected cancer referral within two weeks of visiting the GP. This figure was created by the authors.

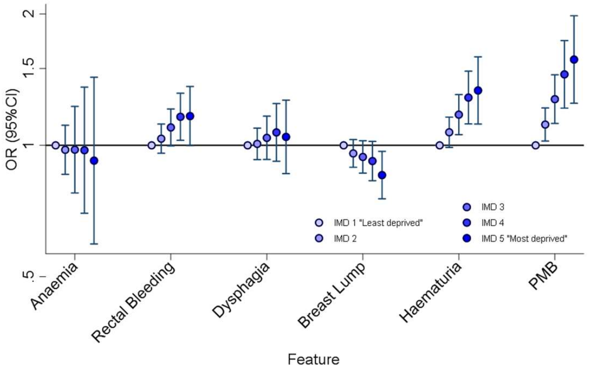

(a) Deprivation

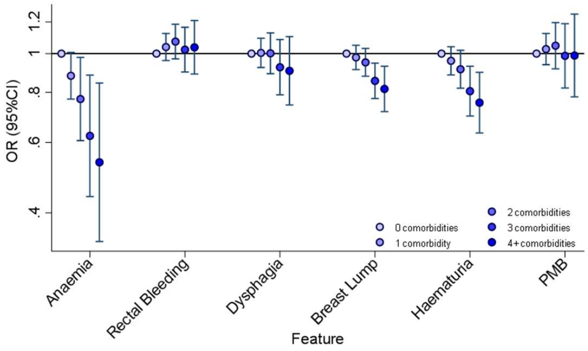

(b) Comorbidities

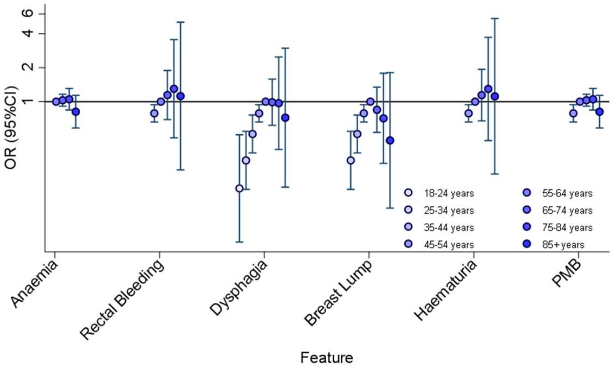

(c) Age

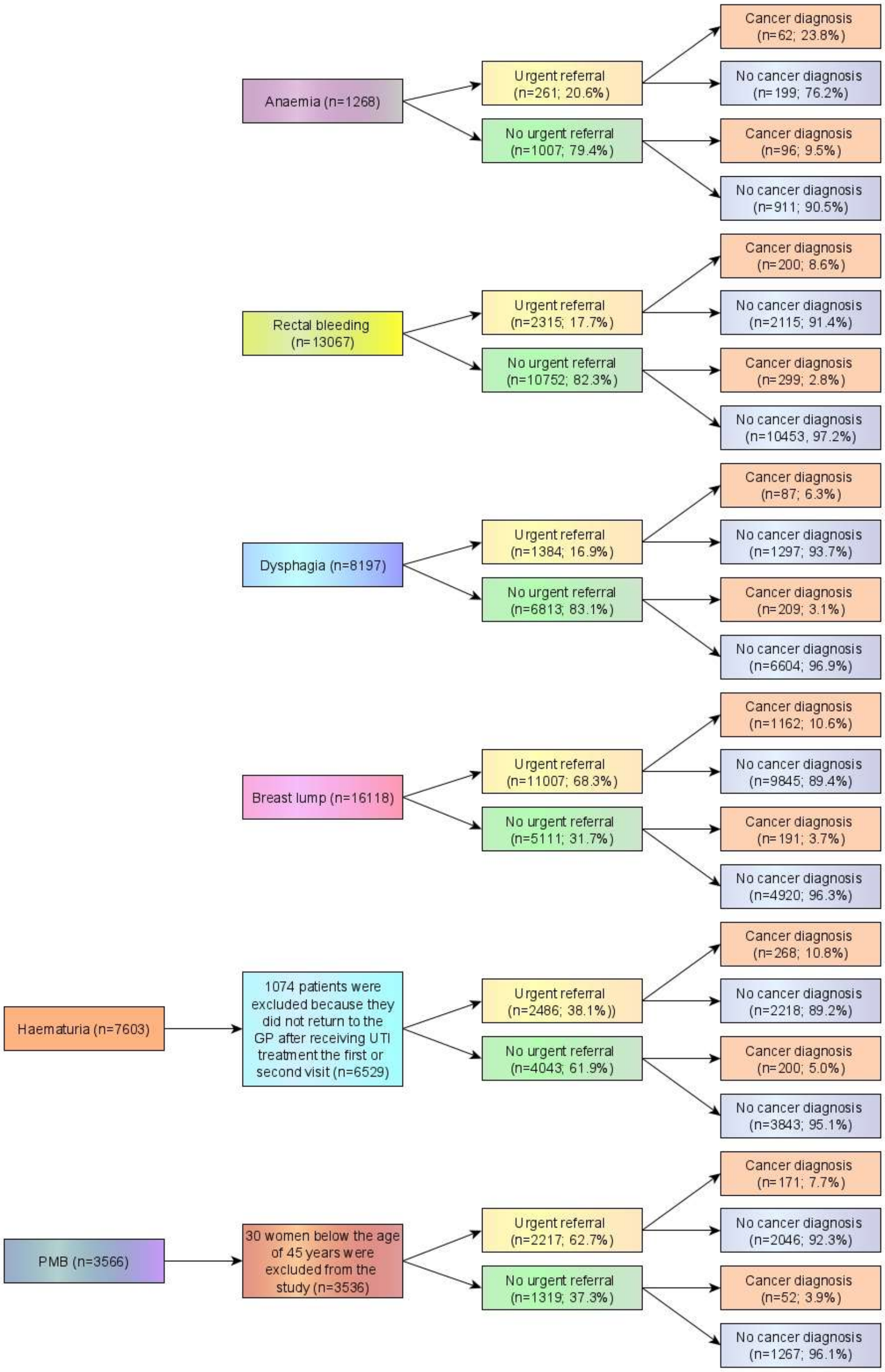

Figure A4. Flowchart of urgent suspected cancer referrals and cancer incidence per feature. This figure was created by the authors.

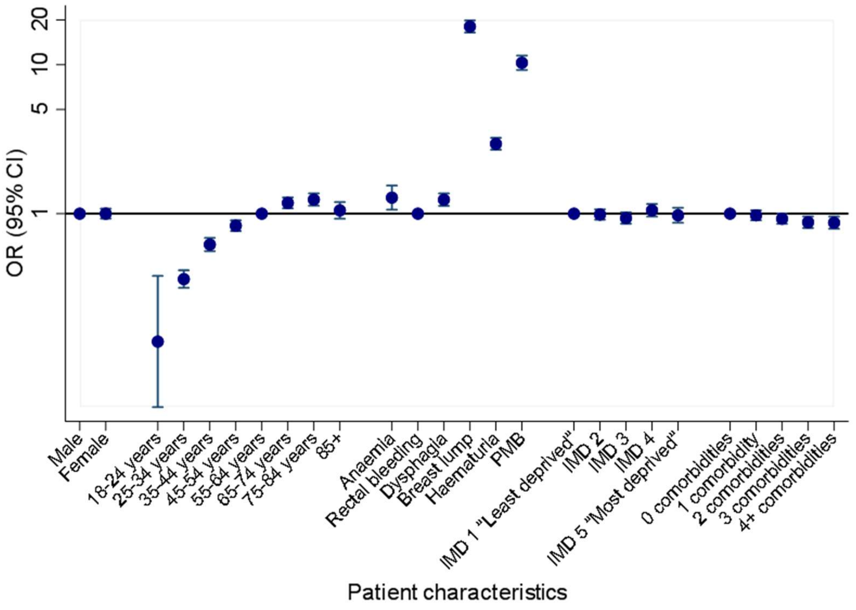

Figure A5. Associations between patient characteristics and receiving a two week wait referral within two weeks of visiting the GP. This figure was created by the authors.

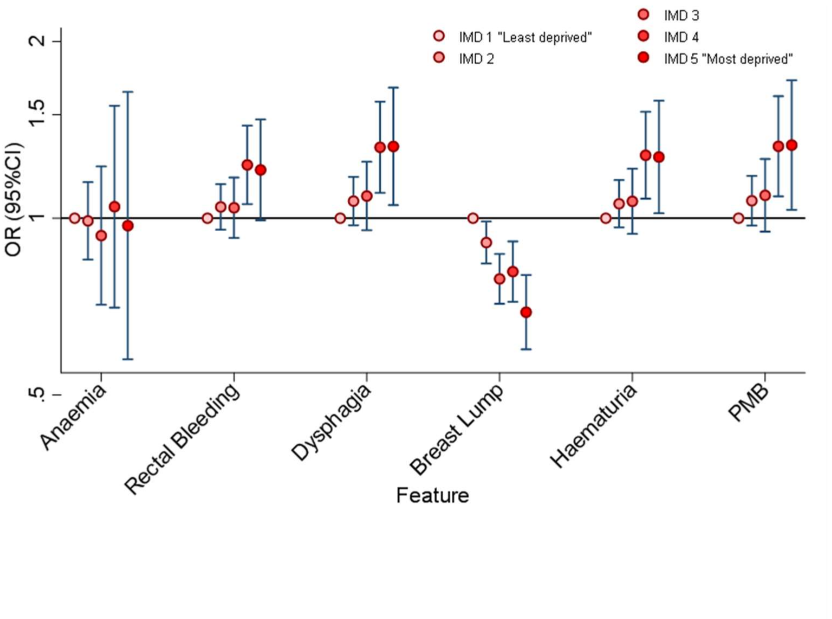

Figure A6. Interactions between deprivation level and feature type and their association with receiving a two week wait referral within two weeks of visiting the GP. This figure was created by the authors.

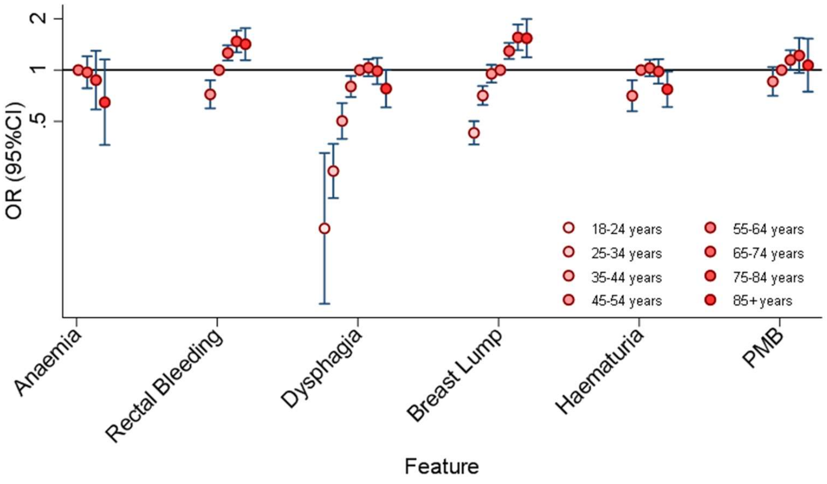

Figure A7. Interactions between age and feature type and their association with receiving a two week wait referral within two weeks of visiting the GP. This figure was created by the authors.
